# Report of Mortality in Buffalo for the Month of June, 1856

**Published:** 1856-08

**Authors:** C. L. Dayton

**Affiliations:** Health Physician


ART. VII.—*Report of Mortality in Buffalo for the month of June*, 1856. By C. L. DAYTON, M. D., Health Physician.

| DISEASES. | No. | Males. | Females. |
|---|---|---|---|
| Accidens, by cars, | 4 | 4 | |
| " Asphyxia Immersorum, | 8 | 7 | 1 |
| " Fracture Cranii, | 1 | 1 | |
| Apoplexia, | 2 | 1 | 1 |
| Ascites, Chronica | 2 | 1 | 1 |
| Cynanche Trachealis, | 2 | | 2 |
| Cancer, | 1 | | 1 |
| Cholera Infantum, | 1 | 1 | |
| Diarrhœa, | 5 | 3 | 2 |
| Debilitas Senilis, | 3 | 1 | 2 |
| Delirium Tremens, | 1 | | 1 |
| Eclampsia, | 9 | 3 | 6 |
| Enteritis, | 1 | 1 | |
| Encephalitis, | 1 | 1 | |
| Febris Scarlatina, | 1 | 1 | |
| " Typhus, | 1 | 1 | |
| " Typhoid, | 2 | 2 | |
| " Congestiva, | 1 | | 1 |
| Hydrocephalus, | 3 | 2 | 1 |
| Ignotus, | 20 | 8 | 4 |
| Morbus Cordis, | 4 | 1 | 3 |
| " Brightii, | 1 | | 1 |
| Marasmus, | 4 | 2 | 2 |
| Morbus Cerebri, | 1 | 1 | |
| Pericarditis, | 1 | 1 | |
| Pneumonitis, | 1 | | 1 |
| Pertussis, | 2 | 2 | |
| Parturitio, | 1 | | 1 |
| Paraplegia, | 1 | | 1 |
| Premature Birth, (Twins,) | 2 | 2 | |
| Paralysis Pulmonalis, (Homœopathist's Diagnosis,) | 1 | | 1 |
| Rheumatism | 1 | 1 | |
| Still-born, | 12 | 3 | 3 |
| Syphilis, Hereditary, | 1 | 1 | |
| Scrophula, | 1 | 1 | |
| Tabes Mesenterica, | 1 | 1 | |
| Tuberculosis Pulmonalis, | 10 | 4 | 6 |
| Total, | 114 | | |

SEXES.

| | |
|---|---|
| Males, | 58 |
| Females, | 42 |
| Sex not given, | 14 |
| Total, | 114 |

REGISTER OF MORTALITY—Continued.

AGES.

| | | | |
|---|---|---|---|
| Still-born, | 12 | Between 10 years and 20 years, | 7 |
| 1 day, | 1 | " 20 " " 30 " | 13 |
| Between 1 day and 30 days, | 5 | " 30 " " 40 " | 12 |
| " 1 month and 6 months, | 8 | " 40 " " 50 " | 6 |
| " 6 months and 12 " | 17 | " 50 " " 60 " | 4 |
| " 1 year " 3 years, | 7 | " 60 " " 70 " | 6 |
| " 3 " " 5 " | 2 | " 70 " " 80 " | 1 |
| " 5 " " 10 " | 2 | " 80 " " 90 " | 1 |
| Total number under 10 years, | 54 | Total number over 10 years, | 50 |

Ages not given,........10. Total,......... 114

NATIVITIES.

| | | | |
|---|---|---|---|
| American, (white, 33) colored, 1, | 34 | Prussian, | 1 |
| German, | 28 | French, | 2 |
| Irish, | 12 | Scotch, | 2 |
| English, | 4 | Pole, | 1 |
| Canadian, | 1 | Country not given, | 29 |

Total,......114

As deaths from paralysis of the lungs is becoming of such alarming frequency in our city, and as it is a disease that very rarely, if ever, comes under the notice of the regular physician, would it not be proper to have a committee of three, or more, appointed to wait upon our neighbors of the small pills and large dilutions, for the purpose of procuring post-mortem specimens exhibiting the pathological condition and morbid anatomy of that organ after being killed by a stroke of paralysis. But, soberly, we must give a certain class of charlatans credit for an amount of honesty that is remarkable; we have reference to the fact, that a large proportion of certificates of cause of death, from that source, are left blank, or filled, unknown, which amounts, in both instances, to a frank acknowledgment of ignorance on that point, that is truly commendable.

